# Myokine myostatin is a novel predictor of one-year radiographic progression in patients with rheumatoid arthritis: A prospective cohort study

**DOI:** 10.3389/fimmu.2022.1005161

**Published:** 2022-10-18

**Authors:** Jian-Zi Lin, Jian-Da Ma, Li-Juan Yang, Yao-Wei Zou, Xue-Pei Zhang, Jie Pan, Qian-Hua Li, Hong-Gui Li, Ze-Hong Yang, Tao Wu, Qian Zhang, Ying-Qian Mo, Lie Dai

**Affiliations:** ^1^ Department of Rheumatology, Sun Yat-Sen Memorial Hospital, Sun Yat-Sen University, Guangzhou, China; ^2^ Department of Radiology, Sun Yat-Sen Memorial Hospital, Sun Yat-Sen University, Guangzhou, China; ^3^ Department of Rheumatology, Shenshan Medical Center, Memorial Hospital of Sun Yat-Sen University, Shanwei, China

**Keywords:** rheumatoid arthritis, myostatin, myopenia, joint destruction, radiographic progression

## Abstract

**Background:**

Associations between rheumatoid arthritis (RA) and reduced skeletal muscle have been studied, and we firstly reported myopenia independently predict one-year radiographic progression in RA. Myokine myostatin can negatively regulate skeletal muscle mass and promote osteoclast differentiation. However, there is no report about their relationships in RA patients. We firstly explored the relationship of serum myostatin and disease characteristics, as well as aggravated joint destruction during one-year follow-up.

**Methods:**

Consecutive RA patients were recruited from a real-world prospective cohort and completed at least one-year follow-up. Baseline serum level of myostatin was measured by enzyme-linked immunosorbent assay. Clinical data in RA patients as well as muscle index in both RA patients and healthy controls were collected. One-year radiographic progression as primary outcome was defined by a change in the total Sharp/van der Heijde modified score ≥0.5 units.

**Results:**

Totally 344 RA patients (age 47.9 ± 12.5 years, 84.0% female) and 118 healthy control subjects (age 42.8 ± 11.3 years, 74.6% female) were recruited. Compared with healthy controls, RA patients showed a higher level of serum myostatin at baseline (3.241 ± 1.679 ng/ml *vs.* 1.717 ± 0.872 ng/ml, *P*<0.001), although lower appendicular skeletal muscle mass index (ASMI, 6.0 ± 0.9 kg/m^2^ *vs*. 6.5 ± 1.0 kg/m^2^, *P*<0.001). In RA patients, those with high myostatin level showed a higher rate of radiographic progression than low myostatin group (45.3% *vs.* 18.6%, *P*<0.001). Furtherly, RA patients were stratified into four subgroups according to serum myostatin and myopenia. Compared with other three subgroups, RA patients with high myostatin overlapping myopenia had the highest rate of radiographic progression (67.2% *vs.* 10.3%-31.4%, *P*<0.001), as well as the lowest proportion of remission and the highest rate of physical dysfunction during one-year follow-up. After adjustment for confounding factors, high serum myostatin (*AOR*=3.451, 95%*CI*: 2.016-5.905) and myopenia (*AOR*=2.387, 95%*CI*: 1.416-4.022) at baseline were risk factors for one-year radiographic progression, especially for those with high myostatin overlapping myopenia (*AOR*=10.425, 95%*CI*: 3.959-27.450) as the highest-risk individuals among four subgroups. Significant synergistic interaction effect was observed between high myostatin and myopenia on one-year radiographic progression (AP=66.3%, 95%*CI*: 43.2%-89.3%).

**Conclusion:**

Myostatin is a novel predictor of aggravated joint destruction in RA patients which has synergistic interaction with myopenia for predicting value.

## Introduction

Rheumatoid arthritis (RA) is a systematic autoimmune disease primarily affecting the joints which is characterized by chronic inflammation and progressive cartilage and bone destruction. Chronic systemic inflammation in RA also leads to extra-articular manifestations such as cardiovascular events, interstitial lung disease, skeletal muscle depletion and so on (1). Recently, the prevalence of reduced skeletal muscle mass in RA patients has been reported to range from 17.1% to 60% (2). Our previous cross-sectional study including 457 RA patients showed 45.1% with myopenia (3), further prospective study of one-year follow-up showed that baseline myopenia independently predict 1-year aggravated joint destruction (4).

A more recent concept proposes that skeletal muscle as an endocrine organ can secret a group of cytokines called myokines. Myokines reportedly exert autocrine functions to regulate muscle metabolism, as well as paracrine/endocrine regulatory functions on distant organs and tissues, including the bone, adipose tissue, brain, and liver (5). Myostatin, a member of the transforming growth factor-β superfamily, is a cytokine produced and released by myocytes that negatively regulating skeletal muscle mass, further supported by animal models evidencing muscle hypertrophy with myostatin gene deletion (6, 7). However, the relationships between serum myostatin and muscle mass and function in previous clinical studies are limited. Myostatin was reported to be associated with sarcopenia in community-dwelling older adults (8). While in patients with cancer cachexia, myostatin concentration was found to be positively correlated with muscle index, muscle density and muscle strength in a cross-sectional study (9). In addition, high expression of myostatin was found in RA synovial tissue (10), and myostatin was reported to be a direct regulator of osteoclast differentiation and its inhibition reduces inflammatory joint destruction in mice (11). However, there is no clinical evidence for the relationships among serum myostatin and joint destruction in RA patients. In this longitudinal study, we prospectively explored the relationship of serum myostatin with RA characteristics and muscle index changes in our RA cohort, and investigated the association between myostatin and myopenia with aggravated joint destruction during one-year follow-up.

## Materials and methods

### Data sources

Consecutive RA patients aged ≥ 16 years and fulfilled the 2010 American College of Rheumatology (ACR)/European League Against Rheumatism (EULAR) classification criteria for RA (12) were recruited between July 2015 to February 2019 from a real-world prospective cohort as described in our previous reports (4, 13). Exclusion criteria were as follows: overlapping other autoimmune diseases (e.g. systemic lupus erythematosus, scleroderma, and dermatomyositis), serious infection, malignancy and pregnancy.

Healthy control subjects were employees or medical students in Sun Yat-sen Memorial Hospital who voluntarily participated in this study from May 2019 to April 2020. The Ethics Committee of Sun Yat-Sen Memorial Hospital approved this study (SYSEC-KY-KS-012 and SYSEC-KY-KS-2022-078), and all subjects signed informed consent forms before clinical data collection.

### Clinical assessments

All patients were treated according to the ACR/EULAR recommendations of ‘treat-to-target’ strategy and completed at least one-year follow up. Available demographic and clinical data were collected at baseline and visited at 3, 6, 9 and 12 months as in our previous reports (4, 13), including age, gender, smoking habits, body mass index (BMI), disease duration, disease activity, physical function, radiographic indicators and medications.

Disease activity defined by clinical disease activity index (CDAI) was divided into four categories: high disease activity (HAD, CDAI >22), moderate disease activity (MDA, 10< CDAI ≤22), low disease activity (LDA, 2.8< CDAI ≤10), and remission (CDAI ≤2.8) (14). Physical dysfunction was defined as Stanford health assessment questionnaire disability index (HAQ-DI) >0 (15).

Conventional radiographs of bilateral hands and wrists (anteroposterior view) were assessed with the Sharp/van der Heijde modified score (16) at baseline and 12 months as we described previously (4, 13). The sum of joint erosion (JE, 0-160) and joint space narrowing (JSN, 0-120) constituted the total modified Sharp score (mTSS, 0-280). The mean intra-class correlation coefficient (ICC) for inter-examiner agreement was 0.957.

Body composition was assessed by bioelectric impedance analysis (InBody 230, Biospace Co., Shanghai, China). Myopenia was defined by appendicular skeletal muscle mass index (ASMI) ≤7.0kg/m^2^ in men and ≤5.7kg/m^2^ in women according to the Asian Working Group for Sarcopenia (17).

### Measurement of serum myostatin

Serum samples from RA patients at baseline and healthy controls were collected after overnight fasting and stored at –80°C. Synovial fluid was synchronously obtained from RA patients with swollen knees. After centrifugation at room temperature (1200 RPM for 5 minutes, 15000 RPM for 10 minutes), the supernatant of synovial fluid was collected and stored at –80°C. The level of myostatin in serum and matched synovial fluid were measured by enzyme-linked immunosorbent assay (R&D Systems, DGDF80-kit).

### Exposure

According to the median level of serum myostatin at baseline, RA patients were divided into low (serum myostatin <2.841ng/ml) or high myostatin groups (serum myostatin ≥2.841ng/ml). The primary exposure was RA patients with high myostatin group, and the second exposure was RA patients with myopenia. Taking them into consideration, RA patients were stratified into four subgroups including low myostatin overlapping non-myopenia group, low myostatin overlapping myopenia group, high myostatin overlapping non-myopenia group, and high myostatin overlapping myopenia group.

### Outcome

The primary outcome was one-year radiographic progression defined as a change in mTSS (ΔmTSS) ≥0.5 units from baseline to 12 months (4).

### Statistical analysis

Statistical analyses were performed with SPSS software for Windows version 25.0 (IBM, Armonk, NY, USA). Data were summarized by means ± standard deviations (SD) or medians with interquartile range (IQR) for continuous variables according to the data distributions and the frequencies with percentages for categorical variables. Two independent samples t-Test or Mann-Whitney test was used to compare the differences of continuous variables according to distributions between two groups. Kruskal–Wallis test was used for comparison in four serum myostatin and myopenia subgroups. Bonferroni correction was used for multiple comparisons in four subgroups. Chi-squared tests or Fisher’s exact tests were used for comparisons of categorical variables among groups. Univariate and multivariate logistic regression analyses by calculating odds ratio (*OR*), adjusted OR (*AOR*) and 95% confidence interval (*CI*) were used to identify the association between exposures and outcome in RA patients. Potential confounders were adjusted including age, gender, smoking habits, BMI, disease duration, rheumatoid factor (RF) status, anti-cyclic citrullinated peptide antibody (ACPA) status, CDAI, HAQ-DI, mTSS at baseline, and one-year cumulative doses of medications. All significance tests were two-tailed and were conducted at the 5% significance level.

To clarify the association between two exposures with outcome, we analyzed the interaction using both the multiplicative and additive scales (18). If the risk of two coexisting risk factors is significantly greater than the product of their independent risks, there is a multiplicative interaction. If the risk of two coexisting risk factors is significantly greater than the sum of their independent risks, there is an additive interaction. To calculate the multiplicative interaction, exposure A × exposure B was included as an independent factor in the logistic regression, and its *OR* and *P* values were used to evaluate the magnitude and significance of the multiplicative interaction. To calculate the additive interaction, we determined the relative excess risk due to interaction (RERI, which represents the relative excess *OR* value caused by the interaction), along with the attributable proportion due to interaction (AP) and the synergy index (S). RERI=(*OR*_AB_−*OR*_A_−*OR*_B_)+1, where *OR*_AB_ is the *OR* of outcome when exposure A and exposure B coexist, *OR*_A_ is the *OR* of outcome when only exposure A exists, and *OR*_B_ is the *OR* of outcome when only exposure B exists. AP=RERI/*OR*_AB_ and S index=[*OR*_AB_−1]/[(*OR*_A_−1)+(*OR*_B_−1)]. RERI and AP are significant if the 95% *CI* does not cross 0, and the S index is significant if the 95% *CI* does not cross 1 (19).

## Results

### Baseline characteristics of the study patients

There were totally 344 RA patients and 118 healthy control subjects recruited (**Table 1**). In total RA group, the average age was 47.9 ± 12.5 years with 84.0% female. The median disease duration was 48 (22-96) months. According to CDAI, there were 20.0%, 28.5%, 29.7% and 21.8% patients in high, moderate, low disease activity and remission, respectively. There were 62.2% patients with physical dysfunction and 21.8% patients without previous glucocorticoid or disease-modifying anti-rheumatic drugs (DMARDs) therapy for at least six months before enrolment (treatment naïve).

**Table 1:** Baseline characteristics of RA patients and healthy controls.

Characteristics	Healthy controls (n=118)	All RA patients (n=344)	*P*
Age, years, mean ± SD	42.8 ± 11.3	47.9 ± 12.5	<0.001
Female, n (%)	88 (74.6)	289 (84.0)	0.022
Active smoking, n (%)	11 (9.3)	52 (15.1)	0.114
BMI, kg/m^2^, mean ± SD	22.7 ± 2.9	21.9 ± 3.3	0.018
ASMI, kg/m^2^, mean ± SD	6.5 ± 1.0	6.0 ± 0.9	<0.001
Myopenia, n (%)	25 (21.2)	152 (44.2)	<0.001
Disease duration, month, median (IQR)	—	48 (22-96)	
Positive RF, n (%)	—	239 (69.5)	
Positive ACPA, n (%)	—	245 (71.2)	
Core disease activity indicators			
28TJC, median (IQR)	—	2 (0-6)	
28SJC, median (IQR)	—	2 (0-5)	
PtGA, cm, median (IQR)	—	3 (1-5)	
PrGA, cm, median (IQR)	—	3 (1-5)	
Pain VAS, cm, median (IQR)	—	2 (1-4)	
ESR, mm/h, median (IQR)	—	32 (15-51)	
CRP, mg/L, median (IQR)	—	5.1 (3.3-20.0)	
DAS28-CRP, median (IQR)	—	3.3 (2.1-4.6)	
DAS28-ESR, median (IQR)	—	3.8 (2.6-5.3)	
SDAI, median (IQR)	—	11.1 (4.3-22.3)	
CDAI, median (IQR)	—	10 (4-20)	
Functional indicator			
HAQ-DI, median (IQR)	—	0.13 (0.00-0.63)	
Physical dysfunction, n (%)	—	214 (62.2)	
Radiographic indicators			
mTSS, median (IQR)	—	9 (2-29)	
JSN subscore, median (IQR)	—	3 (0-13)	
JE subscore, median (IQR)	—	5 (1-17)	
Previous medications			
Treatment naïve^△^, n (%)	—	75 (21.8)	
Glucocorticoid, n (%)	—	178 (51.7)	
csDMARDs, n (%)	—	249 (72.4)	
Biologic agents, n (%)	—	40 (11.6)	

RF, rheumatoid factor; ACPA, Anti-cyclic citrullinated peptide antibody; CRP, C-reactive protein; CDAI, Clinical Disease Activity Index; DAS28-CRP, Disease Activity Score in 28 joints including CRP; ESR, Erythrocyte sedimentation rate; HAQ-DI, Stanford Health Assessment Questionnaire Disability Index; JSN, Joint space narrowing; JE, Joint erosion; mTSS, Modified total Sharp score; PtGA, Patient global assessment of disease activity; PrGA, Provider global assessment of disease activity; Pain VAS, Pain visual analogue scale; RF, Rheumatoid factor; SDAI, Simplified Disease Activity Index; SJC28, 28-joint swollen joint count; TJC28, 28-joint tender joint count. Treatment naive^Δ^, without previous corticosteroids or DMARDs therapy for at least 6 months before enrollment.

In healthy control group (n=118), the mean age was 42.8 ± 11.3 years with 74.6% female. Compared with healthy control subjects, RA patients were older (47.9 ± 12.5 years *vs.* 42.8 ± 11.3 years) with the predominance of female (84.0% *vs.* 74.6%, both *P*<0.05).

### Serum myostatin level at baseline in RA patients

**Figure 1:**
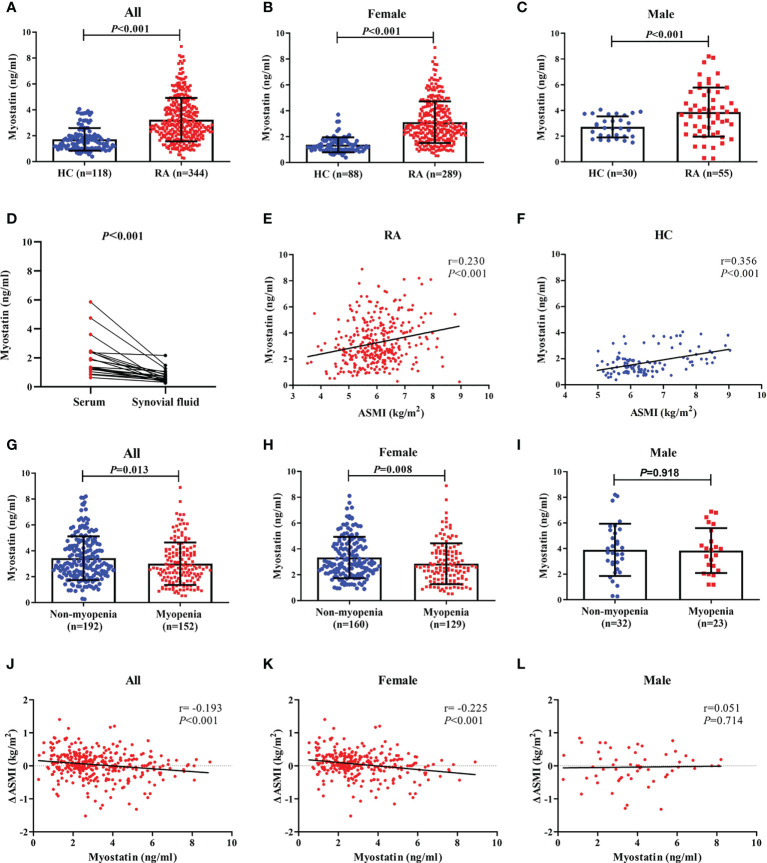
Baseline myostatin level in RA patients and healthy controls. Baseline serum myostatin level in RA patients and healthy controls **(A–C)**, Baseline myostatin of matched serum and synovial fluid from the same RA patients **(D)**, Relationship between serum myostatin and ASMI in all RA patients and healthy controls **(E, F)**, Baseline serum myostatin level in RA patients with myopenia and non-myopenia **(G–I)** Relationship between serum myostatin and ΔASMI in RA patients **(J–L)**. RA, rheumatoid arthritis; HC, healthy controls; ASMI, appendicular skeletal muscle mass index; ΔASMI, a change in ASMI from baseline to 12 months.

Compared with healthy controls, RA patients showed a higher level of serum myostatin at baseline (3.241 ± 1.679 ng/ml *vs.* 1.717 ± 0.872 ng/ml, **Figure 1A**), although lower ASMI (6.0 ± 0.9 kg/m^2^ *vs.* 6.5 ± 1.0 kg/m^2^, both *P*<0.001, **Table 1**). Further gender stratification analysis showed that both female and male RA patients had higher levels of serum myostatin than corresponding healthy controls (female: 3.121 ± 1.607 ng/ml *vs.* 1.376 ± 0.580 ng/ml, **Figure 1B**; male: 3.872 ± 1.910 ng/ml *vs.* 2.717 ± 0.821 ng/ml, **Figure 1C**), although lower ASMI (female: 5.8 ± 0.7 kg/m^2^ *vs.* 6.0 ± 0.5 kg/m^2^; male: 7.0 ± 0.9 kg/m^2^ *vs.* 7.8 ± 0.7 kg/m^2^, all *P*<0.001).

There were 12 RA patients having matched serum and synovial fluid samples. The level of serum myostatin was significantly higher than that of synovial fluid (2.031 ± 1.340 ng/ml *vs.* 0.746 ± 0.475 ng/ml, *P*<0.001, **Figure 1D**).

### Relationship between baseline serum myostatin and disease characteristics

According to the median level of baseline serum myostatin, RA patients were divided into low myostatin group (serum myostatin <2.841ng/ml, n=172) and high myostatin group (serum myostatin ≥2.841ng/ml, n=172). Compared their baseline characteristics, RA patients with high myostatin showed a higher rate of active smoking (20.3% *vs.* 9.9%), higher BMI (22.2 ± 3.4 kg/m^2^ *vs.* 21.5 ± 3.2 kg/m^2^), higher radiographic assessment index including mTSS (median 14 *vs*. 6), JSN (median 5 *vs.* 2) and JE (median 8 *vs.* 4), while lower proportions of female (77.9% *vs.* 90.1%) and previous biologic agents (7.0% *vs.* 16.3%, all *P*<0.05, **Table 2**).

**Table 2:** Comparison of baseline characteristics between RA patients with low and high baseline serum myostatin.

Characteristics	Low myostatin group (n=172)	High myostatin group (n=172)	*P*
Age, years, mean ± SD	47.0 ± 12.8	48.9 ± 12.2	0.181
Female, n (%)	155 (90.1)	134 (77.9)	0.002
Active smoking, n (%)	17 (9.9)	35 (20.3)	0.007
BMI, kg/m^2^, mean ± SD	21.5 ± 3.2	22.2 ± 3.4	0.043
Disease duration, month, median (IQR)	48 (15-89)	52 (24-120)	0.110
Positive RF, n (%)	113 (65.7)	126 (73.3)	0.128
Positive ACPA, n (%)	118 (68.6)	127 (73.8)	0.284
Core disease activity indicators			
28TJC, median (IQR)	2 (0-6)	2 (1-5)	0.985
28SJC, median (IQR)	2 (0-5)	2 (0-4)	0.753
PtGA, median (IQR)	3 (1-5)	3 (1-5)	0.120
PrGA, median (IQR)	2 (1-5)	3 (1-5)	0.124
Pain VAS, median (IQR)	2 (1-4)	2 (2-4)	0.107
ESR (mm/h), median (IQR)	32 (15-53)	32 (18-49)	0.755
CRP (mg/L), median (IQR)	4.0 (3.3-22.1)	6.2 (3.3-19.4)	0.462
DAS28-CRP, median (IQR)	3.3 (2.0-4.7)	3.3 (2.2-4.5)	0.422
DAS28-ESR, median (IQR)	3.7 (2.4-5.3)	3.9 (2.8-5.3)	0.437
SDAI, median (IQR)	11.3 (3.3-22.1)	10.5 (4.9-22.4)	0.471
CDAI, median (IQR)	11 (3-19)	10 (4-20)	0.493
Functional assessment			
HAQ-DI, median (IQR)	0.13 (0.00-0.63)	0.13 (0.00-0.75)	0.590
Functional limitation, n (%)	106 (61.6)	108 (62.8)	0.824
Radiographic assessments			
mTSS, median (IQR)	6 (1-21)	14 (4-40)	<0.001
JSN subscore, median (IQR)	2 (0-8)	5 (1-19)	<0.001
JE subscore, median (IQR)	4 (0-10)	8 (2-22)	<0.001
Previous medications			
Treatment-naive^△^, n (%)	38 (22.1)	37 (21.5)	0.896
Corticosteroids, n (%)	84 (48.8)	94 (54.7)	0.281
csDMARDs, n (%)	126 (73.3)	123 (71.5)	0.718
Biologic agents, n (%)	28 (16.3)	12 (7.0)	0.007

RF, rheumatoid factor; ACPA, Anti-cyclic citrullinated peptide antibody; CRP, C-reactive protein; CDAI, Clinical Disease Activity Index; DAS28-CRP, Disease Activity Score in 28 joints including CRP; ESR, Erythrocyte sedimentation rate; HAQ-DI, Stanford Health Assessment Questionnaire Disability Index; JSN, Joint space narrowing; JE, Joint erosion; mTSS, Modified total Sharp score; PtGA, Patient global assessment of disease activity; PrGA, Provider global assessment of disease activity; Pain VAS, Pain visual analogue scale; RF, Rheumatoid factor; SDAI, Simplified Disease Activity Index; SJC28, 28-joint swollen joint count; TJC28, 28-joint tender joint count. Treatment naive^Δ^, without previous corticosteroids or DMARDs therapy for at least 6 months before enrollment.

During one-year follow-up, compared with low myostatin group, RA patients with high myostatin had significantly higher disease activity indicators at 3, 6, or 9 months including CDAI, 28-joint tender joint count (28TJC), 28-joint swollen joint count (28SJC), patient global assessment of disease activity (PtGA), pain VAS, disease activity score in 28 joints (DAS28) including CRP (DAS28-CRP), DAS28 including ESR (DAS28-ESR), simplified disease activity index (SDAI), and HAQ-DI (all *P*<0.05, **Figures S1A-K**), together with a lower rate of CDAI remission at 9 months (37.8% *vs.* 51.2%, **Figure S1L**), as well as higher proportion of physical dysfunction at 6 months (56.4% *vs.* 40.1%) and 9 months (53.2% *vs.* 34.9%, all *P*<0.05, **Figure S1M**). There was no significant difference between two groups in initial therapy as well as both six-month and one-year cumulative doses of corticosteroids or DMARDs after enrollment (**Table S1**).

**Figure 2:**
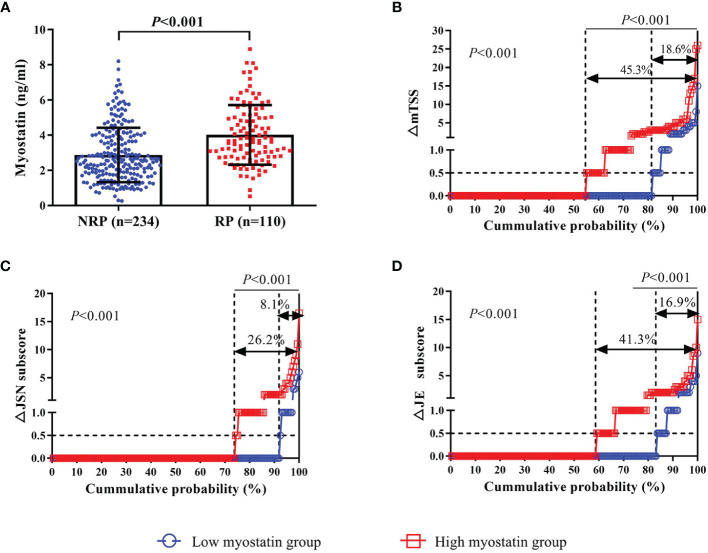
Association between baseline myostatin and one-year radiographic progression. Baseline serum myostatin level in RA patients with or without radiographic progression **(A)**; The cumulative probability distribution of radiographic change including mTSS **(B)**, JSN subscore **(C)** and JE subscore **(D)** from baseline to 12 months in RA patients with or without radiographic progression. NRP, non-radiographic progression; RP, radiographic progression; ΔmTSS, a change in mTSS from baseline to 12 months; ΔJSN subscore, a change in joint space narrowing subscore from baseline to 12 months; ΔJE subscore, a change in erosion subscore from baseline to 12 months.

There were 110 (32.0%) RA patients showing radiographic progression after 12 months. Compared with those without, RA patients with one-year radiographic progression showed a higher level of serum myostatin at baseline (4.017 ± 1.695 ng/ml *vs.* 2.876 ± 1.545 ng/ml, *P*<0.001, **Figure 2A**). Meanwhile, RA patients with high myostatin had a higher rate of radiographic progression (45.3% *vs.* 18.6%, *P*<0.001). The cumulative probability distribution of radiographic change from baseline to 12 months showed that RA patients with high myostatin had higher ΔmTSS, ΔJSN subscore and ΔJE subscore (all *P*<0.01, **Figures 2B-D**).

### Relationship between serum myostatin and muscle mass

Taking the association between serum myostatin and muscle mass into consideration, spearman correlation analysis showed that serum myostatin level was positively correlated with ASMI in both RA patients (r=0.230, *P*<0.001, **Figure 1E**) and control group (r=0.356, *P*<0.001, **Figure 1F**). There were 44.2% RA patients with myopenia. Compared with those without myopenia, RA patients with myopenia showed a lower level of serum myostatin at baseline (3.004 ± 1.640 ng/ml *vs.* 3.428 ± 1.689 ng/ml, *P*=0.013, **Figure 1G**). Further gender stratification analysis showed that female RA patients with myopenia had a lower level of serum myostatin than those without (2.855 ± 1.580 ng/ml *vs.* 3.335 ± 1.601 ng/ml, *P*=0.008, **Figure 1H**), but in male RA patients, there was no difference in serum myostatin between those with or without myopenia (3.839 ± 1.756 ng/ml *vs.* 3.895 ± 2.041 ng/ml, *P*=0.918, **Figure 1I**). Considering the change in ASMI from baseline to 12 months (ΔASMI), spearman correlation analysis showed that baseline serum myostatin level was negatively correlated with ΔASMI in all (r=-0.193, *P*<0.001, **Figure 1J**) and female RA patients (r=-0.225, *P*<0.001, **Figure 1K**), without significant correlation in male RA patients (**Figure 1L**). Compared with low myostatin group, RA patients with high myostatin had significantly lower level of ΔASMI (-0.03 ± 0.41 kg/m^2^ *vs.* 0.09 ± 0.39 kg/m^2^, *P*=0.003), indicating that high serum myostatin at baseline may contribute to reduction of skeletal muscle mass after one-year follow-up.

### Association between serum myostatin and myopenia with disease characteristics

Taking both serum myostatin and myopenia into consideration, RA patients were then stratified into four subgroups including low myostatin overlapping non-myopenia group (n=87), low myostatin overlapping myopenia group (n=85), high myostatin overlapping non-myopenia group (n=105), and high myostatin overlapping myopenia group (n=67). Compared the baseline characteristics with other three subgroups, RA patients with high myostatin overlapping myopenia showed the highest radiographic assessment index including mTSS (median 26 *vs.* 3 *vs.* 10 *vs.* 10), JSN subscore (median 11 *vs.* 0 *vs.* 4 *vs.* 3), JE subscore (median 16 *vs.* 2 *vs.* 5 *vs.* 5), and the highest HAQ-DI (0.3 *vs.* 0.1 *vs.* 0.3 *vs.* 0.1), accompanied with the lowest proportion of female (77.6% *vs.* 89.7% *vs.* 90.6% *vs.*78.1%, all *P*<0.05, **Table 3**).

**Table 3:** Comparisons of baseline characteristics among four RA subgroups by baseline serum myostatin and myopenia.

Baseline characteristics	Low myostatin		High myostatin		
	Non-myopenia(n=87)	Myopenia(n=85)	Non-myopenia(n=105)	Myopenia(n=67)	P*
Age, years, mean ± SD	48 (40 ± 55)	48 (34 ± 59)	49 (40 ± 56)	52 (40 ± 61)	0.250
Female, n (%)	78 (89.7)	77 (90.6)	82 (78.1)	52 (77.6)^†‡^	0.023
Active smoking, n (%)	10 (11.5)	7 (8.2)	19 (18.1)	16 (23.9) ^†‡^	0.033
Disease duration, mo, median (IQR)	37 (12-72)	71 (23-96)	48 (22-116)	66 (27-120)^†^	0.007
Positive RF, n (%)	60 (69.0)	53 (62.4)	79 (75.2)	47 (70.1)	0.295
Positive ACPA, n (%)	67 (77.0)	51 (60.0)	78 (74.3)	49 (73.1)	0.064
Core disease activity indicators					
28TJC, median (IQR)	3 (0-6)	2 (0-7)	2 (0-4)	3 (1-7)	0.305
28SJC, median (IQR)	1 (0-5)	2 (0-5)	1 (0-4)	2 (0-7)	0.592
PtGA, median (IQR)	2 (0-5)	3 (1-5)	3 (1-5)	4 (1-6)	0.120
PrGA, median (IQR)	2 (0-4)	3 (1-5)	3 (1-5)	4 (1-5)	0.099
Pain VAS, median (IQR)	2 (0-4)	2 (2-4)	2 (1-4)	4 (2-5)^†^	0.028
ESR (mm/h), median (IQR)	26 (15-42)	35 (15-68)	32 (19-48)	32 (15-52)	0.414
CRP (mg/L), median (IQR)	3.5 (3.3-12.0)	4.5 (3.3-29.9)	7.2 (3.3-16.7)	4.1 (3.3-22.1)	0.284
DAS28-CRP, median (IQR)	3.0 (1.9-4.7)	3.4 (2.0-4.6)	3.1 (2.2-4.3)	3.7 (2.2-4.9)	0.380
DAS28-ESR, median (IQR)	3.6 (2.4-5.3)	3.9 (2.7-5.3)	3.8 (2.8-4.9)	4.3 (2.6-5.7)	0.532
SDAI, median (IQR)	8.5 (3.3-23.6)	12.3 (4.3-21.0)	9.8 (4.8-20.2)	14.5 (5.0-24.6)	0.329
CDAI, median (IQR)	8 (2-20)	11 (4-18)	9 (4-19)	14 (4-24)	0.343
Functional assessment					
HAQ-DI, median (IQR)	0.1 (0.0-0.4)	0.3 (0.0-0.8)	0.1 (0.0-0.6)	0.3 (0.0-1.0)^†^	0.019
Physical dysfunction, n (%)	48 (55.2)	58 (68.2)	62 (59.0)	46 (68.7)	0.189
Radiographic assessments					
mTSS, median (IQR)	3 (0-10)	10 (2-28)	10 (3-33)	26 (7-61)^†‡$^	<0.001
JSN subscore, median (IQR)	0 (0-4)	4 (1-13)	3 (0-14)	11 (3-29)^†‡$^	<0.001
JE subscore, median (IQR)	2 (0-6)	5 (1-16)	5 (1-18)	16 (5-31) ^†‡$^	<0.001
Previous medications					
Treatment-naive^△^, n (%)	20 (23.0)	18 (21.2)	24 (22.9)	13 (19.4)	0.943
Corticosteroids, n (%)	41 (47.1)	43 (50.6)	54 (51.4)	40 (59.7)	0.477
csDMARDs, n (%)	64 (73.6)	62 (72.9)	78 (74.3)	45 (67.2)	0.759
Biologic agents, n (%)	11 (12.6)	17 (20.0)	5 (4.8)	7 (10.4) ^‡^	0.013

^*^Comparison in four groups by Kruskal–Wallis test.

^†^Compared with low myostatin overlapping non-myopenia patients with Bonferroni correction, *P*< 0.0167.

^‡^Compared with low myostatin overlapping myopenia patients with Bonferroni correction, *P*< 0.0167.

^$^Compared with high myostatin overlapping non-myopenia patients with Bonferroni correction, *P*< 0.0167.

During one-year follow-up, compared with other three subgroups, RA patients with high myostatin overlapping myopenia showed the highest level of DAS28-CRP, SDAI, CDAI and HAQ-DI at 3, 6 and 12 months, together with the lowest proportion of CDAI remission at 3 months (19.4% *vs.* 49.4% *vs.* 37.6% *vs.* 46.7%), 9 months (32.8% *vs.* 54.0% *vs.* 48.2% *vs.* 41.0%) and 12months (28.4% *vs.* 56.3% *vs.* 43.5% *vs.* 47.6%), and the highest proportion of physical dysfunction at 3 months (67.2% *vs.* 34.5% *vs.* 49.4% *vs.* 41.0%), 9 months (62.7% *vs.* 26.4% *vs.* 43.5% *vs.* 45.7%) and 12 months (58.2% *vs.* 26.4% *vs.* 43.5% *vs.* 34.3%, all *P*<0.05, **Figure S2**). There was no significant difference among four subgroups of RA patients in both six-month and one-year cumulative doses of corticosteroids or DMARDs after enrollment, except for high myostatin overlapping myopenia subgroup showing the highest usage proportion of tocilizumab in the initial therapy (28.4% *vs.* 14.9% *vs.* 14.1% *vs.* 12.4%, *P*<0.05, **Table S2**).

**Figure 3:**
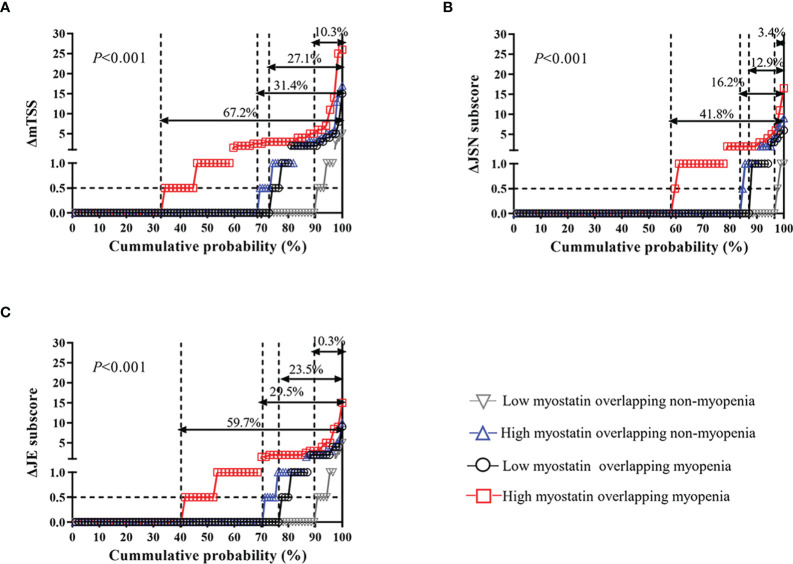
One-year radiographic progression among four RA subgroups by serum myostatin and myopenia at baseline. The cumulative probability distribution of radiographic change including mTSS **(A)**, JSN subscore **(B)** and JE subscore **(C)** from baseline to 12 months in RA patients among four subgroups. ΔmTSS, a change in mTSS from baseline to 12 months; ΔJSN subscore, a change in joint space narrowing subscore from baseline to 12 months; ΔJE subscore, a change in erosion subscore from baseline to 12 months.

Meanwhile, compared with other three subgroups, RA patients with high myostatin overlapping myopenia had the highest rate of radiographic progression (67.2% *vs.* 10.3% *vs.* 27.1% *vs.* 31.4%, *P*<0.001). The cumulative probability distribution of radiographic change from baseline to 12 months showed that RA patients with high myostatin overlapping myopenia had the highest ΔmTSS, ΔJSN subscore and ΔErosion subscore (all *P*<0.01, **Figure 3**).

### Association and interaction between serum myostatin and myopenia with one-year radiographic progression

To investigate the relationships of serum myostatin and myopenia with one-year radiographic progression, univariate and multivariate logistic regression analyses were performed (**Table 4**). Univariate logistic regression analysis showed that as continuous variables, baseline serum myostatin was positively associated with one-year radiographic progression, while baseline ASMI was negatively associated. Meanwhile, as categorical variables, both high myostatin group and myopenia at baseline were predictors of one-year radiographic progression. After adjustment for potential confounders including age, gender, smoking habits, BMI, disease duration, RF status, ACPA status, CDAI, HAQ-DI, mTSS at baseline, and one-year cumulative doses of medications, multivariate logistic regression analysis also showed similar results of baseline serum myostatin (*AOR*=1.534, 95%*CI*: 1.304-1.803), ASMI (*AOR*=0.619, 95%*CI*: 0.431-0.889), high myostatin group (*AOR*=3.451, 95%*CI*: 2.016-5.905) and myopenia (*AOR*=2.387, 95%*CI*: 1.416-4.022, all *P*<0.05, **Table 4**) with one-year radiographic progression.

**Table 4:** Associations and interactions between baseline serum myostatin and myopenia with one-year radiographic progression in RA patients.

Characteristics			Univariate analysis		Multivariate analysis	
			*OR* (95%*CI*)	*P*	*AOR* (95%*CI*)	*P*
Continuous variables						
Myostatin (ng/ml)			1.515 (1.309-1.754)	<0.001	1.534 (1.304-1.803)	<0.001
ASMI (kg/m^2^)			0.568 (0.427-0.757)	<0.001	0.619 (0.431-0.889)	0.009
Multiplicative interaction, myostatin × ASMI (ng/ml × kg/m^2^)			0.768 (0.629-0.937)	0.009	0.703 (0.570-0.868)	0.001
Categorical variables						
High myostatin			3.630 (2.230-5.911)	<0.001	3.451 (2.016-5.905)	<0.001
Myopenia			2.891 (1.810-4.618)	<0.001	2.387 (1.416-4.022)	0.001
Subgroups of serum myostatin and myopenia						
High myostatin	Myopenia	Subgroup				
No	No	Low myostatin overlapping non-myopenia	Ref	NA	Ref	NA
No	Yes	Low myostatin overlapping myopenia	3.215 (1.388-7.445)	0.006	1.432 (0.528-3.887)	0.481
Yes	No	High myostatin overlapping non-myopenia	3.972 (1.778-8.873)	0.001	3.084 (1.314-7.241)	0.010
Yes	Yes	High myostatin overlapping myopenia	17.727 (7.517-41.806)	<0.001	10.425 (3.959-27.450)	<0.001
Multiplicative interaction, high myostatin × myopenia			1.388 (0.478-4.028)	0.546	2.360 (0.720-7.730)	0.156
Additive interaction						
RERI			11.540 (0.277-23.357)	<0.05	6.908 (0.056-13.761)	<0.05
AP			65.1% (42.9%-87.3%)	<0.05	66.3% (43.2%-89.3%)	<0.05
S			3.225 (1.557-6.678)	<0.05	3.745 (1.471-9.534)	<0.05

ASMI, appendicular skeletal muscle mass index; *OR*, odds ratio; *AOR*, adjusted odds ratio; 95%*CI*, 95% confidence interval; Ref, reference; RERI, relative excess risk due to interaction; AP, attributable proportion due to additive interaction; S, synergy index; NA, not applicable.

Multivariate analysis, adjustment for age, gender, smoking habits, BMI, disease duration, RF status, ACPA status, CDAI, HAQ-DI, mTSS at baseline, and one-year cumulative doses of medication.

Furtherly, the interaction between baseline serum myostatin and ASMI as continuous variables was calculated by multiplicative scale, and showed an antagonism effect between them on one-year radiographic progression (*AOR*=0.703, 95%*CI*: 0.570-0.868, *P*=0.001) after adjusting above confounders. For categorical variables, RA patients were divided into four subgroups. Compared with low myostatin overlapping non-myopenia group, the *AOR* of high myostatin overlapping myopenia group was 10.425 (95%*CI*: 3.959-27.450, *P*<0.001), which was much higher than that of high myostatin overlapping non-myopenia group (*AOR*=3.084, 95%*CI*: 1.314-7.241, *P*=0.010) or low myostatin overlapping myopenia group (*AOR*=1.432, 95%*CI*: 0.528-3.887, *P*=0.481) for one-year radiographic progression. The interaction between high myostatin group and myopenia at baseline was calculated by additive and multiplicative scale. In additive scale, the RERI was positive after adjustment (*AOR*=6.908, 95%*CI*: 0.056-13.761), indicating that high myostatin and myopenia at baseline had a significant synergistic effect on one-year radiographic progression. Moreover, AP showed that 66.3% (95%*CI*: 43.2%–89.3%) of the total risk was due to the synergistic interaction between high myostatin and myopenia. In addition, the S index was 3.745 (95%*CI*: 1.471-9.534, all *P*<0.05, **Table 4**), confirming the synergistic interaction between high myostatin and myopenia. While there was no significant multiplicative interaction between them.

**Figure 4:**
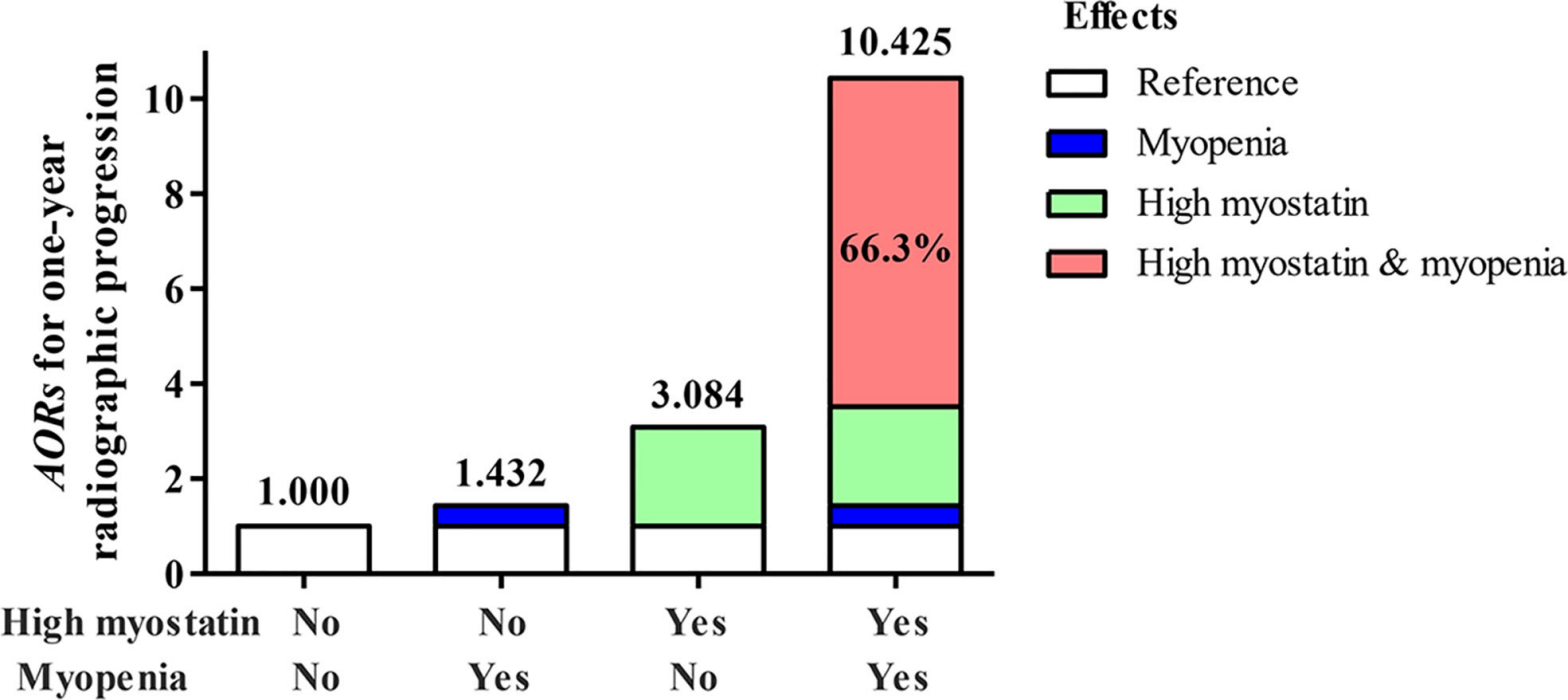
Illustration of the additive synergistic interaction of high myostain and myopenia for one-year radiographic progression. *AOR*, adjusted odds ratio, adjustment for age, gender, smoking, BMI, disease duration, RF status, ACPA status, CDAI, HAQ-DI, mTSS at baseline, and one-year cumulative doses of medications.

To better express these findings, a clear illustration of the results of the additive interaction analyses is presented in **Figure 4**. RA patients with high myostatin overlapping myopenia had a higher *AOR* of one-year radiographic progression than the other subgroups, and 66.3% of the *AOR* in RA patients with high myostatin overlapping myopenia was attributed by the synergistic interaction effect.

## Discussion

This is the first longitudinal study to investigate the relationship between serum myostatin and RA clinical outcomes, and the key finding is that myostatin is a novel predictor of aggravated joint destruction in RA patients. High serum myostatin (*AOR* of 3.451-fold), together with myopenia (*AOR* of 2.387-fold) at baseline were risk factors for one-year radiographic progression, especially for those with high myostatin overlapping myopenia (*OR* of 10.425-fold) as the highest-risk individuals (the rate of one-year radiographic progression 67.2%). There is a synergistic interaction effect between high myostatin and myopenia on one-year radiographic progression with AP 66.3% of its *AOR* attributed by additive interaction.

It has been long known that myostatin acts as myokine, negatively regulates skeletal muscle in humans and animal models. An upregulation of myostatin has been shown in several disease models including cancer cachexia, chemotherapy, kidney failure, heart failure, spinal muscular atrophy, vitamin D deficiency in infantile nephropathic cystinosis, and oculopharyngeal muscular dystrophy (20–22). Previous clinical studies involving the relationship between serum myostatin and muscle mass and function are limited. Plasma myostatin in non-dialysis-dependent patients with chronic kidney disease showed a significant positive relationship with arm lean mass, leg lean mass and trunk lean mass (23). Higher serum levels of myostatin also showed positively correlated with lean mass percentage in women, and better physical fitness including higher number of steps per day and light and moderate-vigorous physical activity in women; greater upper and lower limb strength and endurance in men; and better performance (less time) in the 8-ft timed up-and-go test in both women and men (24). Another study found higher serum myostatin as a significant risk factor of sarcopenia amongst male community-dwelling older adults (8). In our study, compared with healthy controls, RA patients showed a higher level of serum myostatin at baseline in both women and men. The level of serum myostatin was significantly higher than that of synovial fluid in RA patients, and positively correlated with muscle mass both in RA patients and healthy controls. These results suggest serum myostatin in RA may be mainly secreted by muscle tissue rather than joints. Meanwhile, the level of serum myostatin at baseline was negatively correlated with ΔASMI in RA patients after one-year follow-up, which supports the negative regulation role of myostatin on skeletal muscle mass to induce muscle atrophy. These results indicated that myostatin as a myokine may be a double-edged sword in the regulation of muscle mass, which is affected by both aspects of muscle tissue itself and systemic inflammation in RA. Further physiopathologic researches are needed to explore the mechanism of myostatin expression in physiological and pathological states.

Progressive cartilage and bone destruction is one of the most important characteristics of RA. Our previous studies have identified the significant relationship between muscle decrease and joint destruction in RA patients (3, 4). The role of myostatin in muscle growth regulation has been well investigated, and the mechanism by which myostatin regulates bone metabolism is becoming an area of increased interest (25). A study showed that myostatin could inhibit the proliferation and differentiation of mesenchymal stem cells *via* activation of Smad3 and cross-communication of the TGFβ/Smad signal to Wnt/β-catenin/TCF4 pathway (26). Another study showed that myostatin could interfere osteoblastic differentiation by regulating the release of exosome microRNA-218 derived from osteocytes (27). On the other hand, myostatin was identified to directly enhance the ability of osteoclast precursors to differentiate into mature osteoclasts by regulating the expression of NFATC1 through Smad2-dependent signaling pathway (11). Thus, myostatin may serves as a potential anti-osteogenic factor, as well as a direct regulator of osteoclast differentiation in the process of irreversible bone destruction in RA. Using a prospective RA cohort, our data firstly confirmed the role of myostatin on bone destruction in RA patients during one-year follow-up. Higher level of serum myostatin was shown in RA patients with radiographic progression, and high serum myostatin was a risk factor with 3.451-fold for accumulated radiographic destruction in RA patients after adjusting potential confounders.

Taking into consideration that both high serum myostatin and myopenia are related to joint destruction in RA, is there any interaction effect between myostatin and myopenia as myostatin is a key regulator of muscle mass? In our study, there were 19.5% RA patients with high myostatin overlapping myopenia, with the highest rate of one-year radiographic progression (67.2%) than other three subgroups. High myostatin group (3.451-fold) and myopenia (2.387-fold) were both positively associated with radiographic progression. The additive interaction showed that the combination of high myostatin and myopenia had a synergy impact on radiographic progression, which was greater than the sum of the effect of each risk factor. The synergistic interaction effect (6.908-fold) accounted for 66.3% of the total effect (10.425-fold) of the two risks. This is first report of the synergistic effect between high myostatin and myopenia on aggravated joint destruction in RA.

A variety of myostatin inhibitors have been developed for preclinical and clinical studies, including neutralizing antibodies and antagonists of myostatin, myostatin propeptide, decoy myostatin receptor, myostatin targeted siRNA, and antisense oligonucleotides (28). The common effects of these inhibitors include increased muscle mass and strength with decreased fibrosis, and anti-myostatin therapies have been identified effective in several mouse models of muscle wasting associated with cancer and other disorders, even be approved in muscular dystrophies therapy (28, 29). It has been well accepted that the myokines secreted by muscle plays a major role in bone modeling and remodeling (30). Series of studies is actively being examined to explore the relationship of myostatin and bone metabolism. Although a study revealed that the myostatin propeptide treatment could not alter bone formation or bone strength in aged mice (31). Other studies showed that targeting the activation of decoy myostatin receptor ActRIIA could induce bone formation, increasing bone mass and bone strength in ovariectomized rats, and inhibition of ActRIIB in osteoblasts results in increased bone formation in mice (32, 33). In our cohort, elevated serum myostatin was a powerful serological biomarker for cumulative joint destruction in RA, suggesting that targeting myostatin may be a promising treatment option for myopenia and interfering joint destruction in RA.

Previous studies have identified the link between myostatin and inflammation. The levels of serum myostatin and interleukin (IL)-1β were significantly elevated in synovial fluid from RA patients than that from osteoarthritis patients, immunohistochemistry data also revealed a positive correlation between myostatin and IL-1β, TNF-α (10, 34). Additionally, *in vitro* studies performed in human rheumatoid cell line (MH7A) showed that myostatin could increase TNF-α expression *via* the PI3K-Akt-AP-1 signaling pathway, and induce IL-1β expression through ALK receptor, JNK, ERK, and AP-1 signaling pathways, as well as *via* downregulation of miR-21-5p expression (10, 34). Myostatin alone or in combination with IL-17A could enhance the secretion of CCL20 by fibroblasts, contributing to the recruitment of Th17 cells to inflammatory sites and the vicious cycle of inflammation in a mouse model of TNF-α mediated chronic arthritis. Myostatin-deficiency leads to decreased level of the chemokine CCL20 (35). A recent cross-sectional study showed a significant correlation between the elevated serum myostatin and parameters of inflammation including CRP, ESR and DAS28-ESR in RA patients (36). Our cohort study firstly investigated the relationships of serum myostatin and disease activity changes during one-year follow-up, and found higher disease activity indicators at 3, 6, and 9 months and lower rate of CDAI remission at 9 months in RA patients with high myostatin group, though no significant difference at baseline. Furtherly, RA patients with high myostatin overlapping myopenia showed the highest disease activity indicators and lowest rate of CDAI remission at 3, 6, and 12 months than other three subgroups. These results support a link between myostatin and inflammation in RA patients which need more explorations.

There were several limitations in this study. First, this is a single-center study with a small sample size, it would be necessary to carry out further multicenter studies. Second, our study patients recruited only a small proportion of treatment-naïve patients. Although there was no significant difference in medications between RA patients with serum myostatin subgroups or four subgroups by myostatin and myopenia categorization during one-year follow-up, and serum myostatin was still the risk factor of one-year radiographic progression after adjustment for potential confounders, it would be necessary to recruit more treatment-naïve patients and use the same treatment regimen to remove this confounding effect.

In conclusion, in this study, we first reported that elevated serum myostatin was associated with cumulative joint destruction in a real-world RA cohort, proving powerful evidence for the crosstalk between muscle and bone metabolism. Higher serum myostatin and myopenia at baseline have synergistic interaction on predicting one-year radiographic progression in RA patients. Further research on the underlying mechanism is worth exploring in future. As the modulator between muscle and bone metabolism, inhibition of myostatin may be a potential treatment strategy for improving the poor outcomes of RA.

## Data availability statement

The original contributions presented in the study are included in the article/**Supplementary Materials**. Further inquiries can be directed to the corresponding authors.

## Ethics statement

The studies involving human participants were reviewed and approved by The Medical Ethics Committee of Sun Yat-sen Memorial Hospital (SYSEC-KY-KS-012 and SYSEC-KY-KS-2022-078). The patients/participants provided their written informed consent to participate in this study.

## Author contributions

J-ZL and J-DM contributed equally to this work, including conceiving and designing the study, reading and analyzing documents, performing the statistical analysis, and drafting the manuscript. Corresponding author LD and Y-QM also conceived and participated in its design, advised on the search, read and analyzed documents, and edited the paper. L-JY and H-GL participated in myostatin quantitatively measurement of RA patients and healthy controls, and critically revised the manuscript. Y-WZ, X-PZ, JP, TW and QZ participated in clinical assessment and BC measurement of RA patients and healthy controls, and critically revised the manuscript. Q-HL and Z-HY carried out the radiographic assessment and critically revised the manuscript. All authors contributed to the article and approved the submitted version.

## Funding

This work was supported by National Natural Science Foundation of China (grant no. 82171780, 82101892 and 81971527), Guangdong Basic and Applied Basic Research Foundation (grant no. 2022A1515010524, 2020A1515110061 and 2019A1515011928), Project funded by China Postdoctoral Science Foundation (grant no. 2021M703722), Guangdong Medical Scientific Research Foundation (grant no. A2021065), and Guangzhou Municipal Science and Technology Project (grant no. 202102010188).

## Acknowledgments

We thank all patients and medical staff who generously contributed to this study.

## Conflict of interest

The authors declare that the research was conducted in the absence of any commercial or financial relationships that could be construed as a potential conflict of interest.

## Publisher’s note

All claims expressed in this article are solely those of the authors and do not necessarily represent those of their affiliated organizations, or those of the publisher, the editors and the reviewers. Any product that may be evaluated in this article, or claim that may be made by its manufacturer, is not guaranteed or endorsed by the publisher.
