# Supplementary figures and images for: Myokine myostatin is a novel predictor of one-year radiographic progression in patients with rheumatoid arthritis: A prospective cohort study
Source: Front Immunol. 2022 Oct 18;13:1005161. doi: 10.3389/fimmu.2022.1005161 (PMC9623067; doi:10.3389/fimmu.2022.1005161)

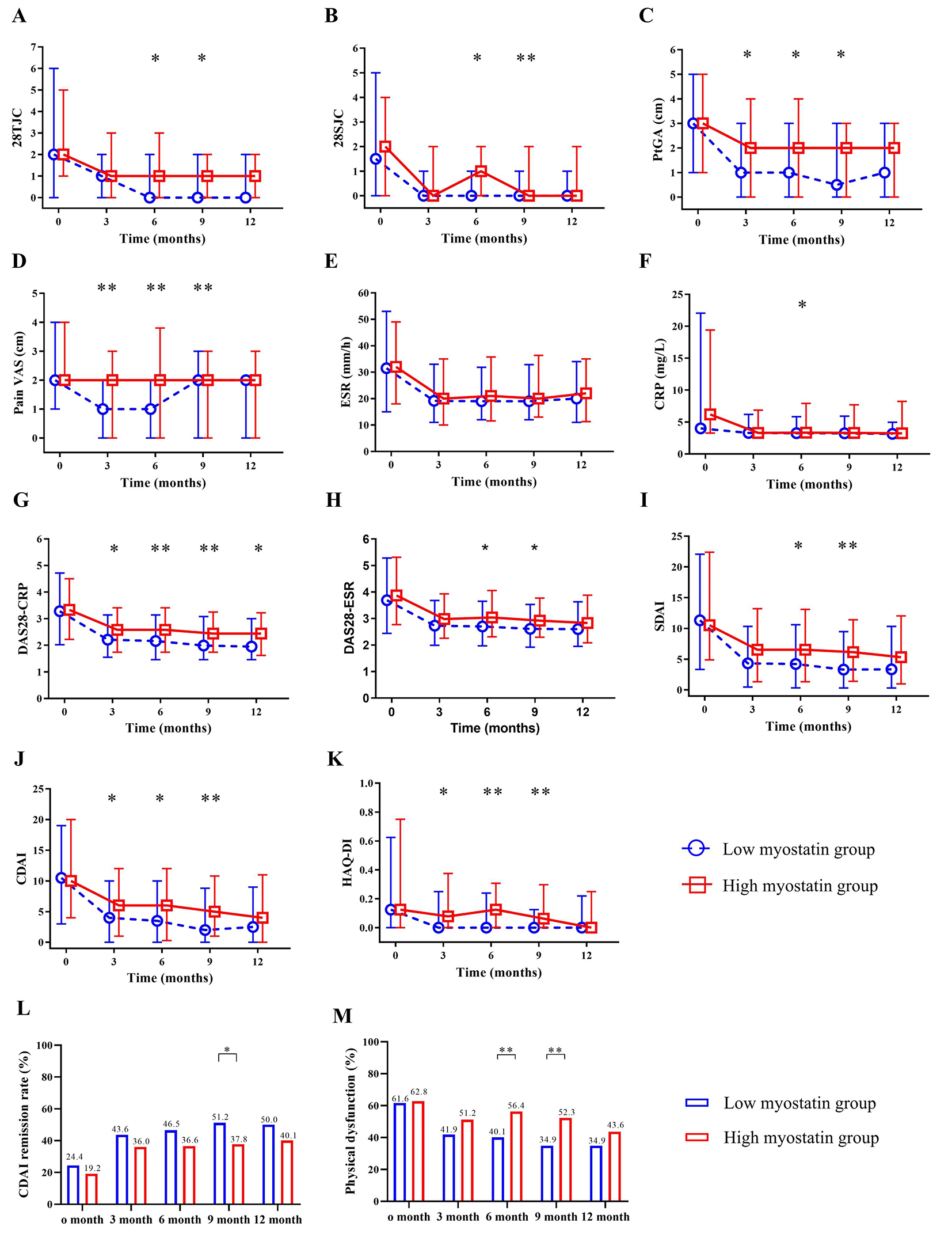

Supplement: Supplementary file 1 [file Image_1.jpg]

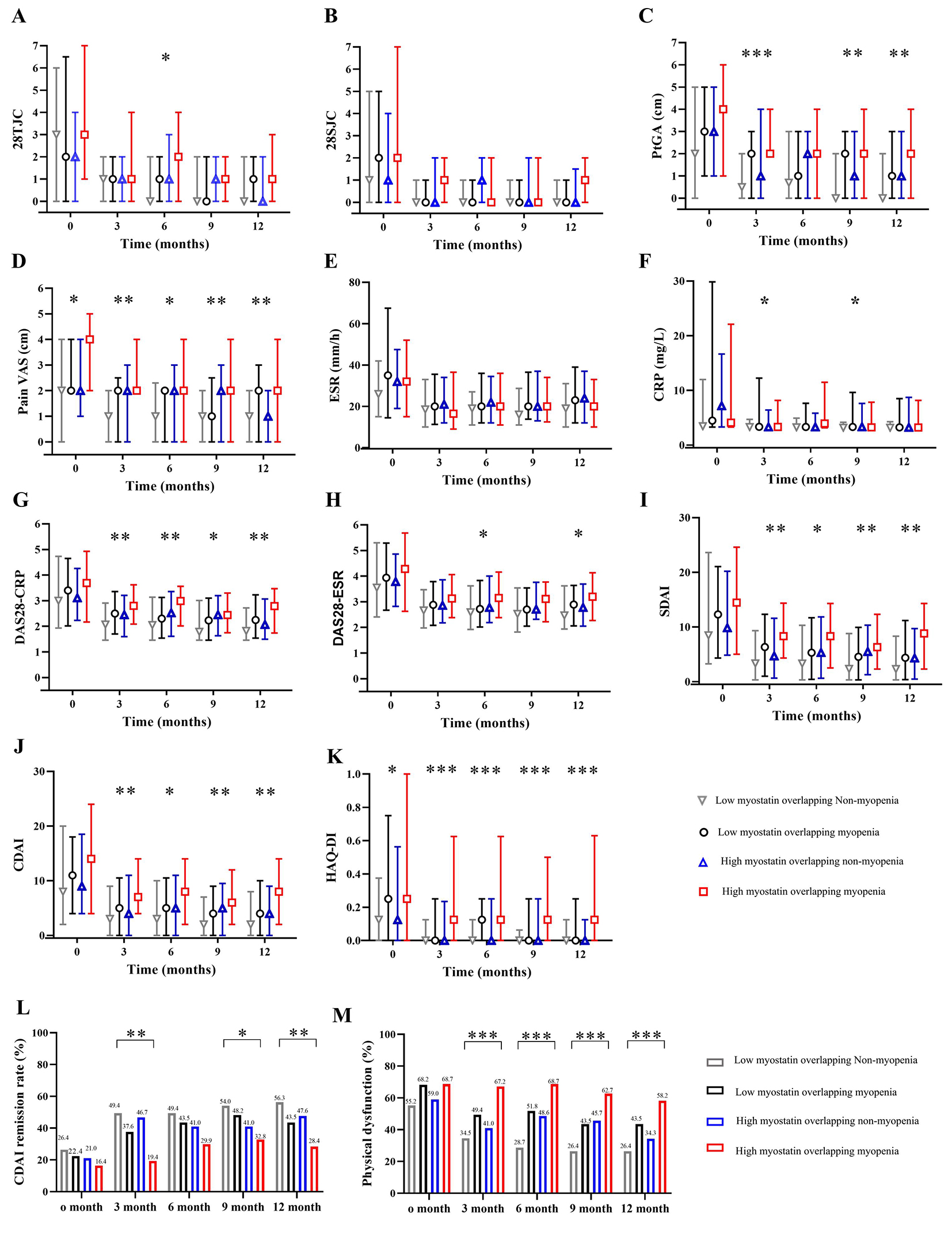

Supplement: Supplementary file 2 [file Image_2.jpg]
