# Supplementary material for: Myokine myostatin is a novel predictor of one-year radiographic progression in patients with rheumatoid arthritis: A prospective cohort study
Source: Front Immunol. 2022 Oct 18;13:1005161. doi: 10.3389/fimmu.2022.1005161 (PMC9623067; doi:10.3389/fimmu.2022.1005161)
Supplement: Supplementary file 3 [file DataSheet_1.doc]

**SUPPLEMENTARY MATERIALS**

**SUPPLEMENT FIGURE CAPTIONS**

**Supplement Figure 1 Comparisons of clinical indicators and therapeutic response during one-year follow-up between RA patients with high and low serum myostatin.**

TJC, tender joint count; SJC, swollen joint count; VAS, visual analogue scale; PtGA, patient global assessment of disease activity; ESR, erythrocyte sedimentation rate; CRP, C-reactive protein; DAS28, Disease Activity Score in 28 joints; SDAI, simplified disease activity index; CDAI, clinical disease activity index; HAQ-DI, Health Assessment Questionnaire Disability Index; CDAI remission, CDAI ≤2.8; Physical dysfunction, HAQ-DI>0. * *P*<0.05, ** *P*<0.01, *** *P*<0.001

**Supplement Figure 2 Comparisons of clinical indicators and therapeutic response during one-year follow-up among four RA subgroups by serum myostatin and myopenia at baseline.**

TJC, tender joint count; SJC, swollen joint count; VAS, visual analogue scale; PtGA, patient global assessment of disease activity; ESR, erythrocyte sedimentation rate; CRP, C-reactive protein; DAS28, Disease Activity Score in 28 joints; SDAI, simplified disease activity index; CDAI, clinical disease activity index; HAQ-DI, Health Assessment Questionnaire Disability Index; CDAI remission, CDAI ≤2.8; Physical dysfunction, HAQ-DI>0. * *P*<0.05, ** *P*<0.01, *** *P*<0.001.

**Table S1 Medications in RA patients with low and high baseline serum myostatin after enrollment**

| **Medications** | **Low myostatin group (n=172)** | **High myostatin group (n=172)** | ***P*** |
| --- | --- | --- | --- |
| Initial medications |  |  |  |
| Glucocorticoids, n (%) | 78 (60.5) | 87 (67.4) | 0.237 |
| Methotrexate, n (%) | 113 (87.6) | 116 (89.9) | 0.554 |
| Leflunomide, n (%) | 83 (64.3) | 91 (70.5) | 0.288 |
| Hydroxychloroquine, n (%) | 52 (40.3) | 40 (31.0) | 0.119 |
| Sulfasalazine, n (%) | 19 (14.7) | 14 (10.9) | 0.351 |
| Cyclosporin A, n (%) | 21 (16.3) | 19 (14.7) | 0.731 |
| Biologic agents, n (%) | 19 (14.7) | 23 (17.8) | 0.500 |
| Tocilizumab, n (%) | 16 (12.4) | 23 (17.8) | 0.224 |
| TNF-α inhibitors, n (%) | 3 (2.3) | 0 (0) | 0.247 |
| Six-month cumulative doses of medications | | | |
| Glucocorticoids, mg, median (IQR) | 544 (0-1200) | 750 (0-1350) | 0.237 |
| Methotrexate, mg, median (IQR) | 260 (260-325) | 260 (260-355) | 0.746 |
| Leflunomide, mg, median (IQR) | 1800 (0-2550) | 1800 (0-2700) | 0.498 |
| Hydroxychloroquine, mg, median (IQR) | 0 (0-36000) | 0 (0-36000) | 0.137 |
| Sulfasalazine, mg, median (5^th^-95^th^ percentile range) | 0 (0-251000) | 0 (0-270000) | 0.431 |
| Cyclosporin A, mg, median (5^th^-95^th^ percentile range) | 0 (0-12525) | 0 (0-12525) | 0.969 |
| Tocilizumab, mg, median (5^th^-95^th^ percentile range) | 0 (0-1200) | 0 (0-2000) | 0.375 |
| TNF-α inhibitors, mg, median (IQR) | NA | NA | NA |
| One-year cumulative doses of medications | | | |
| Glucocorticoids, mg, median (IQR) | 1049 (0-1997) | 1125 (0-2220) | 0.194 |
| Methotrexate, mg, median (IQR) | 520 (480-650) | 525 (520-715) | 0.392 |
| Leflunomide, mg, median (IQR) | 3225 (0-5063) | 3600 (0-5400) | 0.192 |
| Hydroxychloroquine, mg, median (IQR) | 0 (0-72000) | 0 (0-54000) | 0.095 |
| Sulfasalazine, mg, median (5^th^-95^th^ percentile range) | 0 (0-407000) | 0 (0-540000) | 0.834 |
| Cyclosporin A, mg, median (5^th^-95^th^ percentile range) | 0 (0-20100) | 0 (0-27000) | 0.997 |
| Tocilizumab, mg, median (5^th^-95^th^ percentile range) | 0 (0-1552) | 0 (0-2800) | 0.374 |
| TNF-α inhibitors, mg, median (IQR) | NA | NA | NA |

**Table S2 Medications in RA patients of four subgroups after enrollment**

| **Medications** | **Low myostatin** | | **High myostatin** | |  |
| --- | --- | --- | --- | --- | --- |
|  | **Non-myopenia (n=87)** | **Myopenia**  **(n=85)** | **Non-myopenia**  **(n=105)** | **Myopenia**  **(n=67)** | ***P**** |
| Initial medications |  |  |  |  |  |
| Glucocorticoids, n (%) | 51 (58.6) | 56 (65.9) | 73 (69.5) | 46 (68.7) | 0.411 |
| Methotrexate, n (%) | 79 (90.8) | 73 (85.9) | 96 (91.4) | 58 (86.6) | 0.539 |
| Leflunomide, n (%) | 54 (62.1) | 58 (68.2) | 73 (69.5) | 46 (68.7) | 0.705 |
| Hydroxychloroquine, n (%) | 37 (42.5) | 34 (40.0) | 38 (36.2) | 19 (28.4) | 0.304 |
| Sulfasalazine, n (%) | 10 (11.5) | 14 (16.5) | 15 (14.3) | 4 (6.0) | 0.238 |
| Cyclosporin A, n (%) | 12 (13.8) | 13 (15.3) | 14 (13.3) | 11 (16.4) | 0.942 |
| Biologic agents, n (%) | 13 (14.9) | 13 (15.3) | 14 (13.3) | 20 (29.9)^$^ | 0.029 |
| Tocilizumab, n (%) | 13 (14.9) | 12 (14.1) | 13 (12.4) | 19 (28.4)^$^ | 0.035 |
| TNF-α inhibitors, n (%) | 0 (0) | 1 (1.2) | 1 (1.0) | 1 (1.5) | 0.794 |
| Six-month cumulative doses of medications |  |  |  |  |  |
| Glucocorticoids, mg, median (IQR) | 488 (0-1200) | 600 (0-1313) | 750 (0-1350) | 693 (0-1350) | 0.435 |
| Methotrexate, mg, median (IQR) | 260 (260-330) | 260 (260-325) | 260 (260-388) | 260 (260-325) | 0.566 |
| Leflunomide, mg, median (IQR) | 1650 (0-2400) | 1800 (0-2700) | 1800 (0-2700) | 1800 (0-2100) | 0.202 |
| Hydroxychloroquine, mg, median (IQR) | 0 (0-36000) | 0 (0-36000) | 0 (0-36000) | 0 (0-18000) | 0.362 |
| Sulfasalazine, mg, median (5^th^-95^th^ percentile range) | 0 (0-270000) | 0 (0-171000) | 0 (0-270000) | 0 (0.180000) | 0.432 |
| Cyclosporin A, mg, median (5^th^-95^th^ percentile range) | 0 (0-9000) | 0 (0-15600) | 0 (0-13050) | 0 (0-12600) | 0.983 |
| Tocilizumab, mg, median (5^th^-95^th^ percentile range) | 0 (0-1440) | 0 (0-1200) | 0 (0-1368) | 0 (0-2400) | 0.140 |
| TNF-α inhibitors, mg, median (IQR) | 0 (0-0) | 0 (0-0) | 0 (0-0) | 0 (0-0) | 0.247 |
| One-year cumulative doses of medications |  |  |  |  |  |
| Glucocorticoids, mg, median (IQR) | 900 (0-1800) | 1050 (0-2231) | 1050 (0-2156) | 1275 (0-2250) | 0.389 |
| Methotrexate, mg, median (IQR) | 540 (480- 695) | 520 (474-650) | 540 (510-744) | 520 (520-650) | 0.405 |
| Leflunomide, mg, median (IQR) | 2100 (0-4200) | 3000 (0-5400) | 3600 (0-5700) | 3600 (0-4800) | 0.099 |
| Hydroxychloroquine, mg, median (IQR) | 0 (0-72000) | 0 (0-72000) | 0 (0-72000) | 0 (0-42000) | 0.201 |
| Sulfasalazine, mg, median (5^th^-95^th^ percentile range) | 0 (0-522000) | 0 (0-231000) | 0 (0-571500) | 0 (0-360000) | 0.784 |
| Cyclosporin A, mg, median (5^th^-95^th^ percentile range) | 0 (0-18000) | 0 (0-28650) | 0 (0-30000) | 0 (0-25200) | 0.843 |
| Tocilizumab, mg, median (5^th^-95^th^ percentile range) | 0 (0-2256) | 0 (0-1592) | 0 (0-2160) | 0 (0-2800) | 0.096 |
| TNF-α inhibitors, mg, median (IQR) | 0 (0-0) | 0 (0-0) | 0 (0-0) | 0 (0-0) | 0.247 |

^*^Comparison in four groups by Kruskal–Wallis test.

^$^Compared with high myostatin overlapping non-myopenia patients with Bonferroni correction, *P*< 0.0167.
